# Human Exposure through the Diet to Arsenic and Other Toxic Elements: A Literature Review of Scientific Studies Conducted in Catalonia, Spain, in the Current Century

**DOI:** 10.3390/toxics12100749

**Published:** 2024-10-15

**Authors:** Jose L. Domingo

**Affiliations:** Laboratory of Toxicology and Environmental Health, School of Medicine, Universitat Rovira i Virgili, San Llorens 21, 43201 Reus, Spain; joseluis.domingo@urv.cat

**Keywords:** arsenic, heavy metals, dietary intake, human exposure, health risks

## Abstract

Human exposure to arsenic and other toxic elements such as cadmium, lead and mercury may lead to a wide range of adverse health effects. In relation to this, it is well established that the diet is the main route of exposure to both essential and toxic trace elements. In recent years, the levels of toxic elements in foodstuffs have been measured in numerous studies conducted all over the world. Scientific databases show that, in the current century, China and Spain have been the countries where the most surveys on this topic have been carried out. Regarding Spain, Catalonia is the region where most studies aimed at determining the concentrations of trace elements in food have been performed. The objective of this paper was to review the studies carried out in Catalonia on the concentrations of As and toxic metals (including Cd, Hg and Pb) in food, as well as their estimated dietary intakes (EDIs). The results of total diet studies (TDSs) and duplicate diet (DD) studies have been included. For most toxic elements, a continued reduction in the EDI has been observed. This reduction is associated with a decrease in their concentrations in food, and with certain changes in dietary habits. Fish and seafood is the food group showing the highest content of toxic elements. However, none of the adult groups exceeded—in general—the safety thresholds for As, Cd, Hg and Pb established by the European Food Safety Agency (EFSA).

## 1. Introduction

Metals and metalloids are chemical elements, which are found in environmental compartments such as air, soils, vegetation, sediments and waters [1,2,3]. In addition to their natural occurrences, anthropogenic activities like traffic, waste incineration, and emissions of different kinds of industries are also contributing to the increase in their natural levels [4,5,6,7]. Metals/metalloids are environmentally persistent and can bioaccumulate in living organisms. In relation to this, it is well known that various metals/metalloids are essential for several animal species, including humans; other trace elements do not have any known essential function. Regardless, some elements that are considered to be essential for humans may also be toxic, not only at high concentrations but even at rather low levels, depending on the conditions of the exposure. This would be the case for elements such as selenium, iodine, cobalt, iron, zinc, manganese, chromium, molybdenum and copper, which are essential for humans. These elements regulate humoral and cellular immune responses and cellular homeostasis [8,9,10], acting also as cofactors of several enzymes and antioxidant molecules [11,12]. In contrast, other elements, among which arsenic (As), cadmium (Cd), lead (Pb) and mercury (Hg) stand out, do not have any known functionality in the human body and their exposure potentially being toxic, which depends mainly on the level to which one is exposed to [13,14,15,16]. According to the US Agency for Toxic Substances and Disease Registry (ATSDR), these four elements, As, Cd, Hg and Pb, are among the most toxic metals/metalloids with which humans are in contact [17].

In certain conditions, human exposure to As, Cd, Hg and Pb may lead to a wide range of adverse health effects. Thus, the role of Hg and Cd is well known in the development of very serious disorders such as the Minamata and Itai-Itai diseases, respectively [18,19,20]. There may also be increased cancer risks due to chronic exposure to inorganic As and Cd, elements that are classified as “carcinogenic to humans (Group 1)” by the IARC [21]. Moreover, serious hematologic adverse effects, neurological disorders, immunotoxicity and reproductive/developmental toxicity [13,22,23,24,25] are also among the potential health effects caused by chronic/acute exposure to these elements.

Some individuals are exposed to As and toxic metals in the workplace, while others can result from exposure via the inhalation of metal-contaminated air. In turn, dermal exposure to these elements is much less usual—and, consequently, relevant—for public health [26]. For most non-occupationally exposed populations, the dietary intake is the main route of exposure to heavy metals and metalloids [27,28]. Foodstuffs can become contaminated by inorganic and organic pollutants throughout the food chain. Therefore, information about the levels of toxic metals/metalloids in foodstuffs is basic to prevent potential human health risks derived from their intake in the diet. On the other hand, in addition to environmental contamination, some stages of the food chain (i.e., processing and/or packaging), may also contribute, to a greater or lesser extent, to the content of toxic elements in food [29,30].

In recent years, a considerable number of studies aimed at measuring the levels of metals/metalloids in foodstuffs, and to estimate their dietary intakes, have been conducted all over the world. However, most of these surveys have been focused only on some specific food groups (i.e., meats, fish and seafood, milk and dairy products, vegetables, etc.), with the number of total diet studies (TDSs) being much more reduced. Moreover, many surveys have aimed at determining only the levels of one or a few element(s).

According to scientific databases, China and Spain are the countries where more studies on that topic have been carried out in the 21st century. Consequently, the largest number of articles related to this topic are carried out in these countries. In Spain, Catalonia is an autonomous region where most surveys focused on determining the concentrations of heavy metals in food and their intake through the diet have been conducted in this century. Taking this into account, the current paper aimed at reviewing the results of studies carried out in Catalonia on the concentrations of As and toxic metals (mainly Cd, Hg and Pb) in food, as well as to estimate the daily intakes through the diet.

## 2. Search Strategy

Only those studies published in the current century have been included in this review. The databases PubMed (https://pubmed.ncbi.nlm.nih.gov/ (accessed on 2 July 2024) and Scopus (https://www.scopus.com (accessed on 2 July 2024) were used for the search, which included papers published in the period from 1 January 2000 to 5 August 2024. The following terms were used for the search: “arsenic”, “cadmium”, “mercury”, “lead”, “heavy metals”, “food”, “dietary intake”, “tolerable intake”, and “health risks”.

## 3. Human Dietary Exposure to As, Cd, Hg and Pb in Catalonia and Temporal Trends

During the period 2000–2002, Llobet et al. [31] collected samples of foodstuffs that belonged to various food groups. In those samples, the levels of several chemical contaminants were measured. These included organic pollutants (polychlorinated dibenzo-p-dioxins and dibenzofurans furans, polychlorinated biphenyls, polychlorinated naphthalenes, etc.), as well as the potentially toxic elements As, Cd, Hg and Pb. Composite food samples were collected and analyzed for the levels of these four elements. Meat and meat products, fresh fish and shellfish, canned fish, vegetables, fruits, cereals, pulses, milk and dairy products, eggs, fats and oils were the 11 food groups to which the analyzed food samples belonged. The intake of the four toxic elements was estimated for five population groups, according to their age/gender:  children, adolescents, male and female adults, and seniors. The highest levels of the analyzed elements were found in the samples of fish and shellfish, with values of 203.82, 3.33, 8.92 and 4.71 µg/day for As, Cd, Hg and Pb, respectively, which corresponded to a standard adult male of 70 kg body weight (bw). In turn, the lowest As, Cd and Hg levels were found in the pulses, vegetables and fruits, while the lowest Pb concentrations were detected in the pulses and milk, followed by fruits. The highest dietary intakes of As (223.6 μg/day), Cd (15.7 μg/day), Hg (21.2 μg/day) and Pb (28.4 μg/day) corresponded to the group of male adults. When compared with the provisional tolerable weekly intakes (PTWIs) of As, Cd, Hg and Pb, the intakes of these elements for all age/gender groups were below their respective PTWIs. Fish and shellfish was the food group showing the highest contribution to the dietary intake of As, Cd, Hg and Pb.

The study by Llobet et al. [31] had an important limitation. This was the low number of fish species analyzed, which included three species of fresh fish and shellfish, and two species of canned fish. Thus, in order to establish potential recommendations for consumers, the number of analyzed edible marine species was extended in a subsequent survey, in which Falcó et al. [32] measured the levels of As, Cd, Hg and Pb in the most consumed fish and shellfish species in Catalonia. In March–April 2005, composite samples of sardine, anchovy, mackerel, tuna, red mullet, sole, cuttlefish, squid, swordfish, salmon, hake, clam, mussel and shrimp were randomly acquired and subsequently analyzed for the same four toxic elements. For As, the highest level was in red mullet (16.6 μg/g of fresh weight fw), while clam and mussel (0.14 and 0.13 μg/g fw, respectively) were the species showing the highest Cd concentrations. In turn, swordfish (1.93 μg/g fw), mussel and salmon (0.15 and 0.10 μg/g fw), had the highest levels of Hg and Pb, respectively. According to the age/gender groups, the highest intakes of As, Cd and Hg through the consumption of fish and shellfish corresponded to the male seniors: As (217.7 μg/day), Cd (1.34 μg/day) and Pb (2.48 μg/day). For Hg (9.89 μg/day), this corresponded to the group of male adults. The intakes of As, Cd, Pb and total Hg by the population of Catalonia through the consumption of fish and shellfish were again below the respective PTWI values, with the following exception: the estimated intake of methylmercury (MeHg) for boys (1.96 μg/kg body weight/week), which was over its respective PTWI.

In 2006, the temporal trend in the intake of As, Cd, Hg and Pb through the diet of the population of Catalonia was determined [33]. For this, in addition to the group of fish and shellfish, whose samples had been already analyzed by Falcó et al. [32], foodstuffs belonging to the remaining 10 groups that had been included in the initial study [31] were analyzed. The highest levels of As (mean: 4.457 ng/kg fw) and Hg (mean: 0.247 ng/kg fw) corresponded to the samples of fish and shellfish, while those of Cd and Pb were found in pulses (mean: 0.117 ng/kg fw) and in oils and fats (mean: 0.080 ng/kg fw), respectively. The mean levels of As, Cd and Hg were under their respective limits of detection (LODs) for the samples of vegetables, tubers, fruits, eggs, milk and dairy products, and industrial bakery items. Nevertheless, Pb could be detected in the samples of all the food groups. The mean concentrations ranged between 0.012 and 0.080 ng/kg fw for the industrial bakery items and for oils and fats, respectively. The daily dietary intakes of As, Cd, Hg and Pb were the following: 261.01, 9.80, 12.61 and 45.13 μg, respectively. In the initial survey performed by Llobet et al. [31], the daily intakes of As, Cd, Hg and Pb, were 223.59, 15.73, 21.22 and 28.37 μg, respectively. It should be highlighted that in the study by Martí-Cid et al. [33], the non-detected values were assumed to be zero (ND = 0), while in the study by Llobet et al. [31], the non-detected values were considered to be one-half of the respective LODs. Although this last assumption would probably be more realistic, it could add a distortion factor in the comparison of the results, considering, on the other hand, that the analytical procedures and techniques (ICP-MS) used to determine the concentrations of As, Cd, Hg and Pb were always the same. Regardless, the estimated daily intakes (EDIs) of As, Cd, Hg and Pb in the study by Martí-Cid et al. [33] were remarkably lower than the PTWIs of these elements.

To update the human health risks derived from the intake of As, Cd, Hg and Pb through the diet, at the end of 2008, a new collection of food samples was obtained and a new TDS was performed [34]. For each of the 50 analyzed food items, composite samples were made up of 24 individual samples. The selected food items belonged to the same food groups as those analyzed in the previous surveys [31,33]. For the calculations of the dietary intakes of the four toxic elements, when a concentration was under the respective LOD, that value was assumed to be either zero (ND = 0), or equal to one-half of the LOD. With these assumptions, the highest mean levels of As, Cd, Hg and Pb corresponded once again to the group of fish and shellfish. A comparison of these concentrations with those previously reported by Martí-Cid et al. [33], showed increases in the mean levels of As (5.483 vs. 4.457 μg/g), Cd (0.102 vs. 0.034 μg/g) and Pb (0.156 vs. 0.033 μg/g). In contrast, the mean Hg (0.156 vs. 0.247 μg/g) levels decreased. Excluding the fish and shellfish group, the food groups showing the highest mean levels of As, Cd, Hg and Pb followed the following order: cereals, tubers, meat and meat products and vegetables. The dietary intakes (μg/day) of these elements for a male adult of 70 kg bw were the following: 328.37 (total As), 16.22 (inorganic As), 19.47 (Cd), 11.39 (Hg), 10.25 (MeHg) and 101.47 (Pb). In comparison with the EDIs corresponding to the survey performed by Martí-Cid et al. [33], who reported values for As, inorganic As, Cd, Hg, methylmercury and Pb of 261.01, 33.17, 9.80, 12.61, 11.35 and 45.13 μg/day, respectively, the intakes of Cd and Pb increased, while those of inorganic As and MeHg decreased. The observed changes were due to the variations in the levels of these elements in the analyzed foods, but also to certain differences observed in the dietary habits of the population of Catalonia between 2000 and 2008. The EDIs of Cd, Hg and Pb were again lower than their respective PTWIs, that of inorganic As being below its corresponding BMDL_01_. Consequently, at that time, the dietary intakes of As, Cd, Hg and Pb were not associated with health risks for consumers. The same food samples collected for measuring the levels of the four toxic elements were also used to determine the concentrations of other trace elements: Al, Ba, Bi, Cu, Cr, Ge, Mn, Mo, Ni, Sb, Se, Sr and Zn [35]. In general, fish and shellfish, followed by cereals and pulses, were also the food groups showing the highest levels of most of the analyzed elements. However, and even for the fish and shellfish group, the tolerable levels were not exceeded, with only the following exception: Al, whose intake by children slightly exceeded the dietary recommendations. The last TDS on the dietary intake of As, Cd, Hg and Pb in Catalonia was conducted by González et al. [36]. In 2017, samples of most consumed food items in the autonomous community, updated the year (2017) of sampling, were collected to analyze the concentrations of the same four toxic elements. The trends in the risks associated with their intake, 17 years after the first (2000–2002) survey [31], were also determined. For the calculations of the EDIs, when the concentration of an analyzed element was below the detection limit (LOD), that value was considered to be one-half of that LOD. Once again, the maximum mean concentrations corresponded to the fish and seafood group for the total As (3.592 μg/g fw), Cd (0.117 μg/g fw), Hg (0.152 μg/g fw) and MeHg (0.135 μg/g fw). In turn, the highest levels of InAs were found in bread and cereals (0.034 μg/g fw), and those of Pb were detected in chocolate (0.045 μg/g fw). Notable reductions in the intake of the four elements were noted when comparing the levels from the new survey [36] with those from the study conducted by Llobet et al. [31]. The percentage decreases in the daily dietary intakes of total As, Cd, Hg and Pb were 56%, 61%, 70% and 91%, respectively. The intake of InAs was almost identical in the 2012 and 2017 surveys (2.60 and 2.58 μg/day, respectively), while that of MeHg was slightly reduced from 7.33 to 4.49 μg/day. With respect to the potential health risks of the dietary exposure to the four toxic elements, the results of the respective EDIs showed that none of the adult groups exceeded the safety threshold set at that time by the EFSA. However, the intake by toddlers, infants and children exceeded the PTWI for Cd and MeHg [36].

The dietary intakes of As, Cd, Hg and Pb reported in the studies conducted between 2000 and 2017 in Catalonia are summarized in Table 1.

## 4. Concentrations of Toxic Elements in Foodstuffs Purchased from Tarragona County, Catalonia, and Estimated Dietary Intakes

Our laboratory has also carried out various surveys, which were specifically aimed at determining the levels of various trace elements in food samples in a specific area of Catalonia, Tarragona County, where a new hazardous waste incinerator (HWI) was built between 1996 and 1998 [37]. The HWI started its regular operations in 1999. During the period of construction of the HWI, we designed a surveillance program aimed at determining the emissions of As and heavy metals, as well as those of polychlorinated dibenzo-p-dioxins and furans (PCDD/Fs). As a first step of that program, a baseline survey was carried out during the period of construction of the HWI. For the population living near the new facility, the surveillance program also estimated the dietary intakes of PCDD/Fs [38] and the following metals/metalloids: As, Be, Cd, Cr, Hg, Mn, Ni, Pb, Sn, Tl and V [39]. The food items selected for that program belonged to these food groups: vegetables, tubers, fruits, meat and meat products, fish and shellfish, pulses, eggs, cereals, milk and dairy products and sugar. There was a total of 24 different food items. Considering that not all the analyzed foodstuffs, which were among the most consumed by the population in the area, could be of local origin due to production reasons (limitations for meat, milk, fish, etc.), they were randomly purchased from various food stores from Tarragona County, but without taking into account their specific origin. For each food item, 15 individual samples were collected, and composite samples were prepared for the subsequent analyses. For the calculations of the EDIs, when the levels of a metal/metalloid were under the LOD, that value was assumed to be one-half of the respective LOD. In relation to the potentially most toxic elements (As, Cd, Hg and Pb), As could be detected only in the fish and seafood group, as well as in the cereals. The highest levels of Cd were found in the samples of fish and seafood, also being detected in cereals, vegetables and tubers (at similar concentrations). Mercury was detected only in the samples of fish and seafood, while Pb was found at similar levels in all the analyzed samples. For an adult man of 70 kg bw (living in Tarragona County), the EDIs (in μg/day) of As, Cd, Hg and Pb were, respectively, 458, 14.2, 5.3 and 44.9, versus 273, 18.2, 4.8 and 48.6 μg/day, which were the values estimated for As, Cd, Hg and Pb, respectively, in the baseline survey [39]. To investigate the temporal trend in the levels of metals in the food, in July 2006, food samples belonging to the same food groups were again randomly purchased from food stores in Tarragona County [40]. The entire process was like that previously carried out by Bocio et al. [37]. In relation to the levels of As, Cd, Hg and Pb, As could only be detected in the samples of the fish and seafood, cereal and tuber groups. In turn, Cd and Hg were only found in the fish and shellfish samples. Nevertheless, Pb could be detected in the samples of all the food groups, but with two exceptions: milk and dairy products, and eggs. The EDIs of an adult man of 70 kg bw were as follows: 351, 4.6, 7.1 and 39.9 μg/day, for As, Cd, Hg and Pb, respectively. On average, the fish and shellfish group was the main contributor to the intake of As, Cd and Hg through the diet. The same group was also one of the most important for the intake of Pb [40]. As in the two previous surveys conducted in this area [37,39], these intakes were under their respective PTWIs set by the FAO/WHO.

In 2013, the fourth campaign of the food surveillance program linked to the environmental control of the HWI was performed in order to determine the levels of the same elements in samples (composite) of the same food groups [41]. For the adult population living in the area under evaluation, the EDIs (in μg/day) of the toxic elements As, Cd, Hg, and Pb were, respectively, 265, 33.9, 8.5 and 41.7 [41]. These EDIs were similar/lower than those previously reported by Martí-Cid et al. [40]. There was an exception, Hg, whose concentrations progressively and significantly increased with respect to those found in the baseline study [31]. Regardless, these intakes were still below the tolerable limits, except for Cd, whose intake exceeded the PTWI established at that time by the European Food Safety Agency (EFSA) (3.36 vs. 2.5 μg/kg bw/week). The last survey of the surveillance program related to the emissions of the HWI was conducted in 2018 [42]. The concentrations of As, Be, Cd, Cr, Hg, Mn, Ni, Pb, Sn, Tl and V were again determined in foodstuffs purchased from food stores of the same area. The dietary intakes were also estimated. The highest levels of the four toxic elements were found in the samples of fish and seafood: 3.67, 0.074, 0.092 and 0.077 μg/g fw, for As, Cd, Hg and Pb, respectively. Other food groups containing higher levels of these four elements were as follows: cereals for As (0.081 μg/g fw); vegetables and cereals for Cd (0.029 and 0.028 μg/g fw); vegetables (0.078 μg/g fw, similar value to that found in fish and seafood) and oils and fats (0.047 μg/g fw) for Pb, and pulses (0.052 μg/g fw) for Hg, a metal that was not detected in the rest of the food groups. For the adult population of Tarragona County, the EDIs of As, Cd, Hg and Pb were 85.5, 6.55, 2.17 and 21.4 µg/day, respectively, in the last survey. A differential result of that survey concerned nickel (Ni). Among the 11 elements analyzed, in comparison with the concentrations found in the 2013 survey [41], Ni was the only element showing an increase, one that was also noted in its respective EDI. Meat, vegetables and milk were the three main contributors to Ni exposure. Nickel may also be a toxic metal at certain levels and exposure conditions. In relation to this, recently, the EFSA [43] established a tolerable daily intake (TDI) of 13 µg/kg bw for Ni. Thus, according to the results of González et al. [42], the groups of children and adolescents showed a relatively high mean dietary Ni intake (12.82 and 7.10 µg/kg bw/day, respectively). Based on the results on Ni, including this metal in food surveillance programs should be a clear recommendation [42].

Also in Tarragona County, Domingo et al. [44] performed a study aimed at assessing the dietary intake of several metals/metalloids by using a duplicate diet (DD) approach. These data were compared with those derived from previous total diet studies (TDSs) carried out in the same geographical area. Twenty restaurants participate in that survey. Duplicate samples for each meal were collected daily for a total of 10 days from the restaurants over two consecutive weeks (excluding the weekends). The levels of As, Be, Cd, Co, Cr, Cu, Hg, Mn, Ni, Pb, Sb, Sn, Tl, U, V and Zn were measured by ICP-MS. When a concentration was under the LOD, the specific value was assumed to be one-half of the respective LOD. In relation to the daily intake of As, Cd, Hg and Pb, the elements of major concern for the health of the general population, the results of the DD survey showed discrepancies with those previously obtained in our laboratory through a TDS [40]. In the DD study, the intakes of As (199 μg/day) and Pb (19.8 μg/day) were notably lower, while those of Cd (495 μg/day) and Hg (40.4 μg/day) were clearly higher than those found in the TDS. Based on these discrepancies, it was suggested that to compare the results of the different surveys, but also to establish temporal trends in the intake of metals, the use of the same method (either TDS or DD) should be recommended in particular.

## 5. Dietary Exposure to Other Trace Elements

In addition to As, Cd, Hg and Pb, in our laboratory, we have also assessed the exposure to other elements through the diet. Although some of the analyzed elements were essential (cobalt, copper, manganese, etc.), their excess could lead to human health risks. In turn, other non-essential elements in certain amounts might also be toxic (beryllium, vanadium or thallium, for example) [37,40,42]. In 2013, we conducted a specific study aimed at determining the dietary intake of uranium (U) by the population living in Catalonia [45]. With similar methodologies used in our previous surveys, in which other elements were determined [33,34], the levels of U were measured in food items belonging to the 11 food groups that are more consumed by the population of this autonomous community. The highest U levels (0.090 μg/g fw) were also found in fish and seafood, with dairy products being the second most relevant group (0.044 μg/g fw). By contrast, the lowest U concentrations corresponded to the samples of the oil and fat group (0.003 μg/g fw). Nevertheless, U was not detected in the samples of tubers and milk. The dietary intake of U was subsequently estimated for children, adolescents, adults and seniors. The EDI of U for a standard male adult (70 kg bw) was 15.48 μg/day, although the highest EDI corresponded to that by children (20.32 μg U/day). The subgroup of senior females showed the lowest U intake (10.04 μg/day). It was concluded that exposure to U through the diet would not mean health risks for the different age/gender groups of the population.

## 6. Other Studies Conducted in Catalonia on the Levels of As, Cd, Hg and Pb in Food

In 2007, samples of rice, grown in soils that were irrigated with water from the Ebro River, as well as samples of several fruits and vegetables (mandarin, orange, pear, apple, artichoke, tomato, cauliflower and lettuce), were purchased from 16 localities belonging to the riparian zone of the Ebro River in Tarragona Province and its Delta (Mediterranean Sea) [46]. The levels of As, Cd, Cr, Cu, Hg, Mn, Ni and Pb were determined by ICP-OES. Arsenic could only be detected in the samples of rice (0.14 μg/g fw), and in one sample of apple, while Cd and Hg were not detected in any of the samples. However, Pb was found in the samples of mandarin, artichoke, cauliflower, pear and orange (range: 0.01–0.04 μg/g fw). The LODs for these elements were Hg 0.05 μg/g, Cd and Pb 0.03 μg/g and As 0.10 μg/g. The results indicated that in the samples of rice, as well as in those of vegetables and fruits, which grew on the banks of the Ebro River, the levels of As, Cd, Hg and Pb (and those of the remaining analyzed trace elements) were not significantly influenced by the contamination of these elements in the Ebro waters. With respect to the potential health risks for consumers, it was concluded that the exposure to the analyzed metal/metalloids through the consumption of rice and the above indicated vegetables and fruits should not be of concern. In the same area, Nadal et al. [47] assessed the health risks from exposure to various metals through the consumption of several fish and seafood species for the population living near the Ebro River in Catalonia, including the Delta. Among the analyzed species, the highest As concentration was found in cuttlefish (9.12 μg/g fw), sole (6.56 μg/g fw) and hake (6.36 μg/g fw). In turn, mussels showed the greatest Cd level (0.16 μg/g fw). Nevertheless, the only species in which Hg could be detected were sardine and anchovy (both, 0.12 μg/g of fw), while the highest Pb level corresponded to mussels (0.16 μg/g fw). For the population living near the Ebro River in Catalonia, the daily intake of the analyzed toxic elements did not suggest increases in health risks, potentially derived from the consumption of fish and shellfish. The same research group [48] measured the levels of As, Cd, Cr, Cu, Hg, Mn, Ni and Pb in tap water from the same area. The carcinogenic and non-carcinogenic risks were assessed for adults and children. It was found that the concentrations of the analyzed elements with the potential exception of the carcinogenic risks for As would not have a significant impact on the population of the region.

Other studies were carried out in Catalonia during the current century, and were focused on establishing the concentrations of metals in foods and their dietary intake are next discussed. Rodellar-Torras et al. [49] determined the levels of Hg and MeHg in 688 samples of 45 different fish and seafood products commercialized in the city of Barcelona between 2001 and 2007. For the determination of the total mercury, an Advanced Mercury Analyzer AMA254 was used. The limit of quantification was established at 0.10 μg/g. The highest Hg levels corresponded to these two species: a porbeagle (*Lamna nasus*) and a little tunny (*Euthynnus quadripunctatus*), with means of 7.6 and 1.4 μg/g, respectively. In the rest of the samples, the Hg levels ranged between 0 and 0.98 μg/g. Based on those results, the average weekly intake of total mercury for the Catalan population was found to be 0.783 µg/kg bw. This value was well below the recommended PTWI (5 µg/kg bw) at that time. On the other hand, Cano-Sancho et al. [50] characterized the bioaccessibility of As, Hg and MeHg in samples of swordfish, tuna, mackerel, sardine, seabream, monkfish, common sole, mussel, prawn and cuttlefish. All of these species are of frequent consumption in Catalonia. All the samples were grilled before being analyzed. The bioaccessible fraction of As ranged between 72% and 89% for mackerel and sardine, respectively. The bioaccessible fraction of Hg was lower, at 77% and 61% for cuttlefish and monkfish, respectively, while most of the remaining species showed fractions lower than 50%. In turn, MeHg was only detected in two species: swordfish and tuna, for which the highest levels corresponded to the cooked samples. In these two species, tuna and swordfish, after in vitro digestion the fractions released to the gastrointestinal juice, were 42% and 57%, respectively. While the As risks are not generally overestimated, there could be an overestimation of the health risks for consumers with respect to Hg and MeHg. This takes into account that both Hg and MeHg showed a lower and variable bioaccessibility in marine species. Recently, to investigate the concentrations of total Hg in fish, Capodiferro et al. [51] carried out a large survey, in which 1345 specimens corresponding to 58 different species of wild fish and seafood from the western Mediterranean Sea, including the coasts of Catalonia, were analyzed. Another objective of that study was to define the species that would meet the current EU recommendation for human exposure to Hg (0.5 μg Hg/g fw) derived from the consumption of fish and seafood. The total mercury was determined using an automated atomic absorption spectrometer. The LOD was 0.0009 μg/g (dry weight). Only certain species did not exceed the value of 0.5 μg Hg/g fw in the analyzed specimens. The following species were considered to be safe: seabream, pearly razorfish, salema, common dolphinfish, sardines, anchovies, blue whiting, picarel, blackspot seabream, gilthead, surmullet, painted comber, brown meagre, and squid. It was concluded that if the these species are consumed as the only source of fish, this could equate to weekly intakes in the range of 49–70% of the recommended current PTWI of MeHg, in the worst scenario. The median Hg levels ranged between 0.1 μg/g fw in salema (*Sarpa salpa*) and 3.0 μg/g fw in porbeagle *(Lamna nasus*).

## 7. Discussion, Conclusions and Future Directions

First of all, regarding the measurements of the levels of trace elements in the studies reviewed above, the analytical techniques used have been inductively coupled plasma mass spectrometry (ICP-MS) and atomic absorption spectrometry (AAS)/graphite furnace. Also, cold vapor atomic absorption spectrometry has been occasionally used for specific analyses of mercury. The differences in the analytical methods/techniques in the studies reviewed here may have had a notable impact on the results reported. This means there are certain limitations on drawing general conclusions, taking into account that the methodologies used in the studies examined here could have been notably different. All of the articles included in this review have been published in peer-reviewed journals. Therefore, it was assumed that the analytical methods/techniques used to measure the concentrations of metals/metalloids in foodstuffs were adequate. This should have been verified by the reviewers (and editors) of the respective journals in which the studies were published.

Since 2000 and until 2017, total diet studies (TDSs) have been periodically performed in Catalonia, Spain. They were aimed at measuring the concentrations of various organic contaminants, as well the levels of four toxic elements (As, Cd, Hg and Pb) in the most consumed foodstuffs in this autonomous community. For both the organic and inorganic contaminants, the dietary intakes were also estimated, and the temporal trends were established. The current review has been focused on As and toxic metals, mainly Cd, Hg and Pb, but it also includes some information on other potentially toxic metals such as Ni and U. The results of the TDSs reported in scientific articles have been summarized and discussed, while other studies conducted also in Catalonia during the 21st century have also been included in this review.

With respect to the results of the TDSs, it has been noted that the exposure to As, Cd, Hg and Pb in human diets has been remarkably reduced over the course of 17 years [36]. This notable reduction is associated not only with a decrease in the concentrations of these elements in food, but also with certain changes in the dietary habits of the population of Catalonia. Specifically, for a standard male adult of 70 kg bw, the safety thresholds established by the EFSA were not exceeded for any of the four toxic elements. With respect to other age/gender groups, adolescents (10–17 years) had the highest intake of Cd, Hg and Pb. This group was also the second most important for As exposure, the group of children aged 1–3 years being the first. Nevertheless, if the daily intake is considered in terms of body weight (bw), logically, toddlers and children show the highest intakes of the four elements. These intakes exceeded, in some cases, the PTWI established by the EFSA. Thus, the dietary intake of MeHg by children aged 1–3 years (2.23 μg/kg bw/week) was higher than the maximum recommended value (1.3 μg/kg bw/week). Something similar also occurred for Cd, with infants (2.69 μg/kg bw/week) slightly exceeding the PTWI set by the EFSA (2.5 μg/kg bw/week). Since the initial TDS [31], the fish and seafood group has always been the main contributor to the dietary intakes of toxic elements. Specifically, in the last TDS, cuttlefish and hake were the species with the most important contribution to the excess Cd and MeHg intake by infants [36]. Although this is an experimental result, obviously, by itself, it cannot represent a sufficient reason to recommend a reduction in the consumption of fish by children in general, or even that of these two species, in particular.

In relation to the dietary intake of As, Cd, Hg and Pb (and other metals such as Ni and U), the results of the rest of the studies conducted during the current century in Catalonia have not raised special concern for the people living in this region. The results of various surveys, the samplings in which were carried out in different geographical areas of the autonomous community, and which have included different food items, have shown similar conclusions. In general, the dietary intake of As and the analyzed heavy metals should not equate to health risks for the non-occupationally exposed population. Although fish and seafood are the main contributors to the intake of As, Cd, Hg and Pb, only those individuals who frequently consume high quantities of certain species could be significantly increasing their potential health risk due to excessive exposure to As and heavy metals. The results of the studies conducted in Catalonia during the current century have not raised concerns about the health risks caused by dietary exposure to the analyzed elements.

Spain and China are the countries where most studies on the topic of the present review have been conducted in this century. With respect to Spain, in addition to Catalonia, a notable number of surveys have also been carried out in the Canary Islands, another Spanish autonomous community. The “Grupo Interuniversitario de Toxicología Ambiental y Seguridad de los Alimentos y Medicamentos” of the University of La Laguna has been especially active in this area. In recent years, the dietary intake of Cd, Hg and Pb by the population of the Canary Islands was estimated by that research group [52,53,54]. These authors have also evaluated the human exposure to various toxic metals derived from the consumption of some specific foods such as cereals, soybean beverages, canned mushrooms, edible seaweed, as well as certain fish species [55,56,57,58,59,60,61]. Other researchers in the Canary Islands have also measured the concentrations of heavy metals in edible species of fish and shellfish, while the human dietary exposure to toxic elements was also estimated [62,63,64,65]. Other studies conducted in the current century in various Spanish regions are also available in the scientific literature [66,67,68,69,70,71,72,73,74]. However, they are not specifically discussed here, taking into account that this review was focused only on studies carried out in Catalonia.

Doubtless, a comparison of the results obtained in the surveys conducted in different Spanish regions with those from the studies carried out in Catalonia should be an issue of interest. However, considering the important differences in the materials and methods that were used in each of these studies, in particular, the different food items that were analyzed, the number of samples, the analytical techniques, etc., the comparison of the results would certainly be very complicated, if not impossible. Despite this, there is a common denominator in the results of most studies. Thus, there is an important coincidence in the high percentage of contribution to the total dietary intake of As and heavy metals made by fish and shellfish. Despite this indisputable fact, the health benefits of regular consumption of fish and shellfish generally outweighs the potential adverse effects of environmental contaminants in general, and those of As, Cd, Hg and Pb, specifically [75]. Notwithstanding, some specific groups in the population, such as children, women of childbearing age, and pregnant women, as well as breastfeeding mothers, should be especially careful with their diets in order to avoid potential health risks due to exposure to chemical contaminants through food. Of course, this includes As and toxic heavy metals.

On the other hand, another coincidence seems to be the general trend towards a decline over time in the concentrations of metals in food, and therefore, in the human dietary intake of these contaminants. Due to the current environmental policies in most countries, continued reductions in the environmental concentrations of As and heavy metals has already happened and it is expected to continue to happen to an even greater extent. In the coming years, this should mean lower human exposure to toxic elements from all sources in general, and from the diet in particular. Regardless, from a public health point of view, it is highly recommended that the potential health risks of dietary exposure to toxic elements are periodically assessed, and their temporal trends are established.

## Figures and Tables

**Table 1:** Dietary intakes of As, Cd, Hg and Pb determined through total diet studies (TDSs) conducted in Catalonia, Spain, in the 21st century.

Period of Sampling	Analyzed Food Groups	Food Groups/Food Items with the Highest Levels of As, Cd, Hg and Pb	Dietary Intake	Remarks	References
2000–2002	Meat and meat products, fresh fish and shellfish, canned fish, vegetables, fruits, cereals, pulses, milk and dairy products, eggs, and fats and oils.	Fish and shellfish, with intakes of 203.82, 3.33, 8.92 and 4.71 µg/day, for As, Cd, Hg and Pb, respectively, for an adult male of 70 kg body weight	For adult males: As (223.6 μg/day), Cd (15.7 μg/day), Hg (21.2 μg/day) and Pb (28.4 μg/day)	The dietary intakes of the four elements were, for all age/gender groups of the population of Catalonia, below their respective PTWIs.	Llobet et al. [31]
2005	The 14 most consumed (in Catalonia) species of fish and shellfish.	For As, red mullet (16.6 μg/g of fresh weight fw); for Cd, clam and mussel (0.14 and 0.13 μg/g fw, respectively). In turn, swordfish (1.93 μg/g fw), and mussel and salmon (0.15 and 0.10 μg/g fw) showed the highest levels of Hg and Pb, respectively.	The highest intakes through consumption of fish and shellfish were those of As (217.7 μg/day), Cd (1.34 μg/day) and Pb (2.48 μg/day) for male seniors. For Hg (9.89 μg/day), this was for the group of male adults.	Only the estimated intake of methylmercury for boys (1.96 μg/kg bw/week) was over the PTWI.	Falcó et al. [32]
2006	The same food groups already analyzed in the Llobet et al. [31] TDS, with the exception of fish and shellfish, assessed by Falcó et al. [32].	Taking together the results with those of Falcó et al. [32], it was noted that the highest levels of As and Hg corresponded to those from fish and shellfish, while those of Cd and Pb were detected in pulses (Cd) and oils and fats (Pb).	The dietary intakes of As, Cd, Hg and Pb were 261.01, 9.80, 12.61 and 45.13 μg/day, respectively.	In this study, the non-detected values were assumed to be zero (ND = 0), while in the study by Llobet et al. [31], the non-detected values were considered to be one-half of their respective LODs (ND = 1/2 LOD). This could explain some of the differences in the results.	Martí-Cid et al. [33]
2008	The same food groups as those of the previous surveys [31,32,33].	The highest mean levels again corresponded to fish and shellfish with decreases (in comparison to the values reported by Martí-Cid et al. [33]) for As (5.483 vs. 4.457 μg/g), Cd (0.102 vs. 0.034 μg/g) and Pb (0.156 vs. 0.033 μg/g), and an increase in the mean Hg (0.156 vs. 0.247 μg/g) level.	The dietary intakes (μg/day) of these elements for a male adult of 70 kg bw were the following: 328.37 (total As), 16.22 (inorganic As), 19.47 (Cd), 11.39 (Hg), 10.25 (MeHg) and 101.47 (Pb).	Compared with the previous TDS [33], the intakes of Cd and Pb increased, while those of inorganic As and methymercury decreased.	Martorell et al. [34]
2017	Meat and meat products, fish and seafood, vegetables, tubers, fruits, eggs, milk and dairy products, bread and cereals, pulses, oils, industrial bakery, sauces, chocolates and infant food.	Maximum mean values found in fish and seafood for total As (3.592 μg/g fw), Cd (0.117 μg/g fw), Hg (0.152 μg/g fw) and MeHg (0.135 μg/g fw). The highest levels of InAs were found in bread and cereals (0.034 μg/g fw), and those of Pb in chocolate (0.045 μg/g fw).	The dietary intakes (μg/day) for total As, InAs, Cd, Hg, MeHg and Pb were 98.2, 2.58, 6.12, 6.36, 4.49 and 2.62, respectively.	The dietary exposure of As, Cd, Hg and Pb notably decreased between 2000 and 2017. The decreases in the food concentrations of these elements, and certain changes in the dietary habits, were key factors for the observed reductions.	González et al. [36]

## Data Availability

Data will be made available on request.
